# Impact of virtual supervised tooth brushing on caries experience and quality of life among primary school children: study protocol for a randomized controlled trial

**DOI:** 10.1186/s13063-023-07111-8

**Published:** 2023-02-20

**Authors:** Haya Alayadi, Areej Alsiwat, Haifa AlAkeel, Munirah Alaskar, Maram Alwadi, Wael Sabbah

**Affiliations:** 1 Dental Biomaterials Research Chair, Dental Health Department, College of Applied Medical Sciences, King Saud University, Riyadh, Saudi Arabia; 2 Dental Services Development Team, Dental Services Department, Riyadh Second Health Cluster, Ministry of Health, Riyadh, Saudi Arabia; 3 Dental Health Department, College of Applied Medical Sciences, King Saud University, Riyadh, Saudi Arabia; 4 Faculty of Dentistry, Oral & Craniofacial Sciences, King’s College London, London, UK

**Keywords:** Tooth brushing, Randomized controlled trials, Quality of life, Dental caries, Dental care for children

## Abstract

**Background:**

Dental caries is one of the most common diseases affecting children world widely as well as in the Kingdom of Saudi Arabia. Supervised tooth brushing programs are implemented throughout the world to provide young children’s developing teeth with additional fluoride as a form of dental caries prevention. While school-based supervised tooth brushing programs have been proven to improve young children’s oral health, virtual supervised teeth brushing programs have not been assessed. The purpose of this protocol is to assess the impact of virtual supervised tooth brushing on caries experience and quality of life among primary school students in Riyadh, Saudi Arabia.

**Methods:**

This is a cluster randomized controlled trial comparing a virtual supervised tooth brushing program against no intervention applied. A total of 1192 (596 in each group) 8–9-year-old children in Riyadh primary schools, Saudi Arabia, will be recruited for the trial. Schools (cluster) will be randomly selected and allocated to either group. Clinical assessment for caries experience will be conducted in six points (baseline, + 3 months, + 6 months, + 12 months, + 24 months, + 36 months) by dental hygienists using the World Health Organization criteria. Data on sociodemographic behavioral factors and children’s quality of life will be collected with every clinical assessment through a structured questionnaire. The primary outcome is the change in caries experience (the number of teeth with untreated dental caries, filled and missing teeth) in both primary and permanent teeth over 36 months.

**Discussion:**

Virtual education as well as some health consultation through the pandemic period had enabled an effective IT infrastructure in Saudi Arabia. Virtual supervised tooth brushing is a proposed initiative. It is also an opportunity for targeting a large portion of the population with a high level of disease as a quarter of the Saudi population is younger than 15 years. This project should provide high level evidence on the effectiveness of virtual supervised tooth brushing. The findings should potentially inform policies related to the continuation/implementation of school-based programs in Saudi Arabia.

**Trial registration:**

ClinicalTrials.gov, ID: NCT05217316. Registered on 19 January 2022.

## Background

Dental caries is one of the most prevalent diseases in the world, which affects an estimated 621 million children worldwide and reaches its peak prevalence at the age of 6 [1]. In the Kingdom of Saudi Arabia (KSA), several reviews have been conducted to determine the prevalence of dental caries in the Kingdom, which concluded that it is highly prevalent among Saudi children [2–5]. The prevalence of dental caries in primary teeth ranges from 21 to 100%, according to a recent systematic study published in 2021 [4], indicating that dental caries remains one of the most common chronic diseases among children in KSA.

Children who have dental caries experience pain, lack of sleep, difficulties speaking and eating, and missed school time [6, 7]. Children’s general health and quality of life can also be impacted by dental caries, which can interfere with their ability to eat well, attend school, and perform well in class. In a recent systematic review, it was found that children with one or more decaying teeth were more likely than those without evident caries to exhibit low academic performance and poor attendance [8]. Children who have healthy mouths, however, can eat, speak, and interact with others without experiencing any pain, discomfort, or embarrassment [1, 9].

Caries is largely preventable [10]. Brushing teeth with fluoride toothpaste is one of the most effective preventive methods, as evidence has shown that fluoride toothpaste reduces caries rate by (24%) [11, 12]. Using toothpaste is the best approach to provide fluoride to the tooth surface since it immediately coats the tooth surface while brushing removes plaque. Other tooth brushing-related parameters, such as frequency of brushing [13], the quantity of toothpaste used [14], length of time spent brushing teeth [14], and tooth brushing supervision [11], may also affect the efficacy of fluoride toothpaste on children’s oral health.

Supervision by adults while the children brush their teeth can ensure that they have enough exposure to fluoride toothpaste each day [11]. There may also be unintended educational consequences. Children that are supervised might learn how to brush their teeth more effectively [15]. Children might place more priority on oral health and develop better oral health behaviors in general. They might also enhance the quantity and frequency of daily tooth brushing. Finally, surveillance while brushing may have an impact on children through a form of prolonged Hawthorne effect [16], causing them to change their oral health self-care behaviors in reaction to the knowledge of being watched.

Supervised tooth brushing programs are implemented throughout the world to provide young children’s developing teeth with additional fluoride as a form of dental caries prevention. Numerous published research studies have demonstrated the value of supervised tooth brushing programs in schools [15, 17–21]. For instance, Clark et al. (2019) [17] studied children from Northland, New Zealand, aged 10 to 13, and found that those who received the supervised school tooth brushing intervention had significantly reduced caries prevalence than those who did not. This is a quasi-experimental study. At baseline, all children received routine dental examinations, including bitewing radiographs and the ICDAS to record dental caries. A supervised tooth brushing program was implemented for half of the children. Exams were given again at the end of the school year. There were 335 children at the start, with 240 (71.6%) being followed up on. Caries was found in 7.3% of the tooth brushing group and 71.5% of the control group. Similarly, a randomized controlled trial recruiting primary school children in Northwest London compared the variances in caries increment between a group that received teacher-supervised tooth brushing with a fluoride toothpaste at 1450 parts per million (ppm) once a day at school and a non-intervention group. It concluded that the intervention group had a statistically significant lower caries incidence compared to the control group (2.60 versus 2.92; 10.9%; *p*-value < 0.001) [18]. These findings suggest that implementing preventative measures in schools can be an efficient approach to improving oral health.

Virtually supervised school tooth brushing is being developed as the world’s perspective on education shifts due to COVID-19 lockdown, replacing old teaching techniques with more modern ones. Virtually supervised tooth brushing is a proposed initiative in KSA, where schools are run virtually and have IT infrastructure that has proven to be beneficial. While teeth brushing under supervision has been proven to be effective, virtual tooth brushing has never been tested. A recent study examined the effectiveness of virtual oral hygiene instruction against traditional one to raise school health mentors’ knowledge levels and their impact on primary school students’ plaque index (PI). Both kinds of education were found to significantly improve students’ PI scores, but virtual instruction was found to be more successful. This may raise the question of whether virtual teeth brushing under supervision has a comparable impact [22]. Consequently, this study aims to assess the impact of virtual supervised tooth brushing on caries experience and quality of life among primary school children in Riyadh, KSA.

## Aim

The aim is to assess the effectiveness of virtual supervised tooth brushing on primary school children’s caries experience in Riyadh, Saudi Arabia.

## Objectives

To examine the difference between two intervention groups in:

1. Changes in caries experience—decayed (untreated dental caries), missing, filled teeth in primary and permanent teeth, plaque level, and gingival condition—upon the follow-up time among primary school children
2. Changes in the frequency of brushing per day upon the follow-up time among primary school children
3. Positive change in the impact of oral health on the child’s daily life (QoL)

## Methods and design

This manuscript adheres to the Standard Protocol Items: Recommendations for Interventional Trials (SPIRIT) 2013 Statement [23] for reporting of protocols for clinical trials and the Consolidated Standards of Reporting Trials (CONSORT) 2010 Statement—extension for cluster randomized trials [24].

### Study design and setting

The trial is a cluster randomized control trial, assessor-blinded running in two parallel groups with a 1:1 allocation ratio. The trial will be conducted among selected primary schools in Riyadh city, Saudi Arabia, over 3 years duration. Randomization will be at cluster level (school) to prevent any contamination probability between participants across intervention groups if located in the same school.

### Setting and participants

The trial will be conducted across 20 primary schools in Riyadh city, Saudi Arabia. A list of all governmental primary schools in Riyadh city, Saudi Arabia, will be obtained from the Ministry of Education as the study is stratified based on the area of residence (affluent/non-affluent), and random selection will be conducted. Schools with in-house dental clinics and participating in any dental screening/preventive program will be excluded from the trial.

Children in selected primary schools will be considered eligible to join the trial if they meet all inclusion criteria and none of the exclusion criteria.

Inclusion criteria

- Children aged 8 to 9 years (4th grade at school) at baseline
- Both Saudis and non-Saudis
- Children for whom the person with parental responsibility has signed the consent/assent form

Exclusion criteria

- Children in 5th and 6th grade (11–12-year-olds) as they would have left schools by the time of the follow-up assessment (18 months later)
- Children who refuse to participate in the study
- Special need schools are excluded

### Trial interventions

Oral epidemiological assessment will be conducted in-person in the preventive dentistry clinics to all children within both groups (intervention and control group). The difference between the two groups lies in that the intervention group will receive virtual supervision on tooth brushing once a week by the dental hygienist through “Mawid” a Ministry of Health (MOH) Central Appointment System and for 4 days during the week they are provided with different videos of the correct tooth brushing method. The video will be sent via “SMS” to the parents of students on a daily basis; thus, a reminder was not considered. This continues for three consecutive months.

Appointments will be booked via “Mawid” with is a (MOH) Central Appointment System and mobile application for managing appointments in primary healthcare centers and hospitals nationwide [25], while the virtual appointments will be conducted using “Sehhaty,” a public mobile application integrated with “Mawid” used for various health services such as sick leave, e-prescription, and managing in-person and virtual appointments nationwide [26]. All virtual appointments during the study will be booked accordingly and subjects will receive an SMS with the date and time of his/her appointment and a link on how to use the service [27]. The virtual appointments will be conducted using the collaboration of two national platforms. “Sehhaty” is to be used by the subjects. And “ANAT” is to be used by the physicians, whereas “ANAT” is an MOH-approved platform used for virtual consultations booked in both “Mawid” and “Sehhaty” [28].

A comparison against a control with no intervention was not considered due to ethical concerns (children would be examined but not informed about their oral findings). Thus, children in both groups will be informed about their oral findings and will be educated about how to make an appointment through “Sehhaty” application; all treatment will be provided free of charge. It is worth noting that both groups will receive fluoride toothpaste (1450 ppm) along with the examination received. In this trial, we will consider those with untreated dental caries as screened positive children, whereas all others will be considered as a screened negative children, based on the World Health Organization (WHO) criteria [29]. At the time of examination, parents or caregivers will be provided an electronic WHO questionnaire to filled out [29].

**Figure 1:**
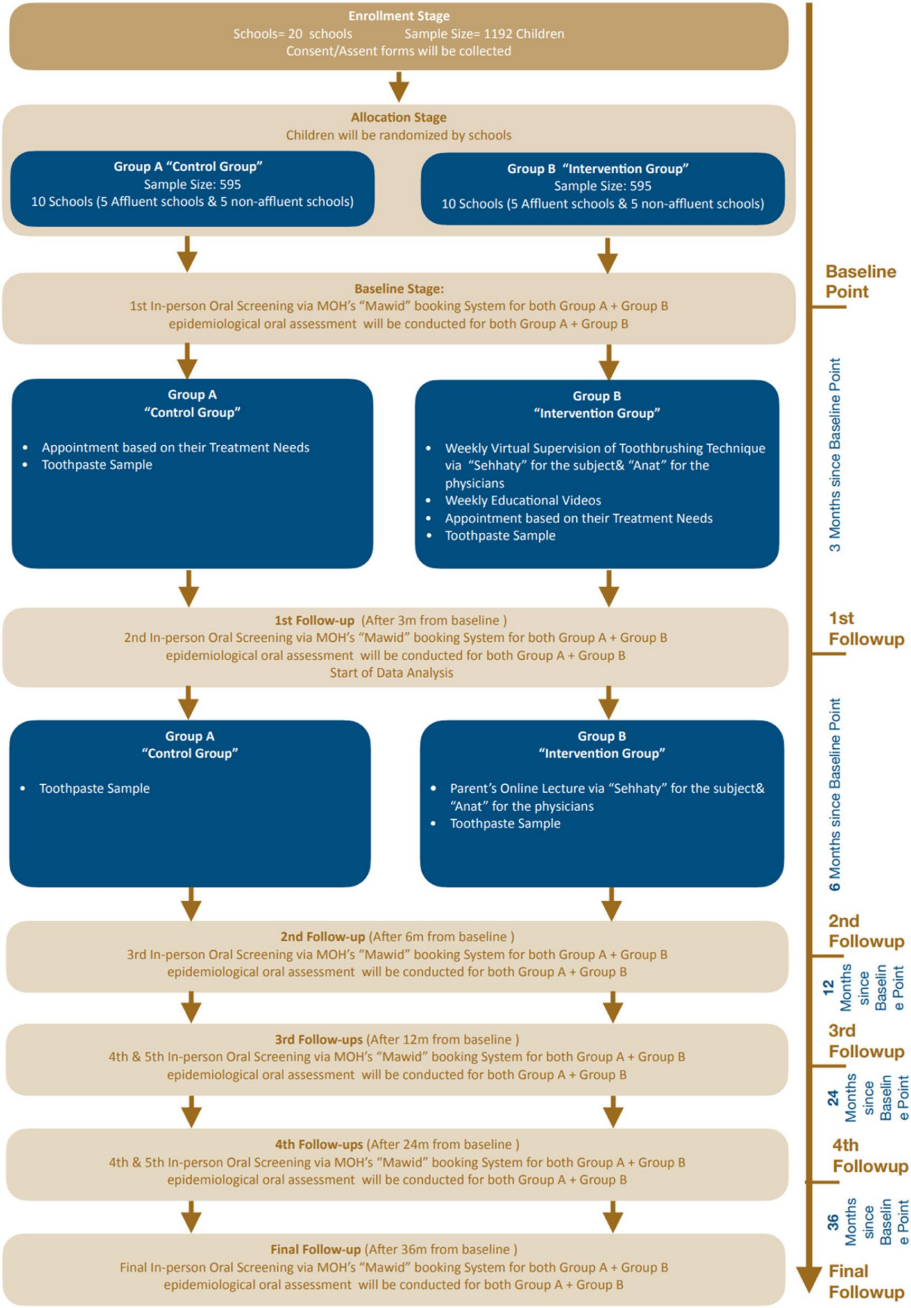
Flowchart of the project process

The intervention group will receive virtual supervision on tooth brushing once a week by the dental hygienist through “Mawid,” a Ministry of health (MOH) Central Appointment System, and for 4 days during the week, they are provided with different videos of the correct tooth brushing method via “SMS” sent to the students’ parents. This continues for three consecutive months. The only difference between intervention and control groups rely on that the control group will not receive any virtual supervision on tooth brushing appointments. Figure 1 illustrates the flow chart of the project intervention.

### Outcomes

The combination of self-reported and clinical outcomes robust the findings on the effectiveness of virtual supervised tooth brushing. The clinical measure will be a blinded outcome measures (caries experience), plaque, and gingival scores as the examiners are blinded to the groups the children are assigned to. Unlike the clinical outcome, the self-reported frequency of tooth brushing per day and the impact of oral health on the child’s daily life (QoL) are considered a subjective measure.

#### Primary outcome

The study’s primary outcome is the change in caries experience—the number of decayed (untreated dental caries) missing and filled teeth—in both primary and permanent teeth over 36 months. The underlining assumption of the selected primary outcome is that children in the intervention group will be exposed to more fluoride (toothpaste daily exposure) in addition to mechanical tooth cleaning (correct dental plaque debridement through correct brushing technique) during the trial period leading to a fewer incidence of dental caries than those in the control group.

#### Secondary outcomes

Two secondary outcomes for this trial include the following: the changes in plaque and gingival score based on Leo and silence plaque and gingival index [30] and the change in the frequency of tooth brushing per day and the perceived oral health on child’s daily life (as reported by the parents).

### Trial procedure

The time schedule of the enrolment assessment, intervention, and assessment is shown in Table 1.

**Table 1:** The project time schedule of enrolment, intervention and assessment

						Study period		
	Enrolment	Allocation	Post allocation					Close-out
**Timepoint**	*-3-0 months*	*0 months*	*0 months*	*3 months*	*6 months*	*12 months*	*24 months*	*36 months*
**Enrolment:**								
Permission	**X**							
Sending paperwork to parents	**X**							
Training and calibration of examiners	**X**							
Randomisation		**X**						
Obtaining parental consent / child assent		**X**						
Eligibility screening for participants		**X**						
**Intervention:**								
**Intervention arm**: Exposed to daily unified tooth brushing technique videos 5/days			**X**	**X**				
**Control arm:** No intervention			**X**	**X**				
**Assessment (both intervention arms)**								
Parental Questionnaire: Sociodemographic data	**X**		**X**	**X**	**X**	**X**	**X**	**X**
Parental Questionnaire: Behavioural data	**X**		**X**	**X**	**X**	**X**	**X**	**X**
Primary outcome: clinical examination (untreated dental caries)			**X**	**X**	**X**	**X**	**X**	**X**
Secondary outcome: clinical examination (gingival and plaque score)			**X**	**X**	**X**	**X**	**X**	**X**
Secondary outcome: frequency of daily toothbrushing	**X**		**X**	**X**	**X**	**X**	**X**	**X**
Secondary outcome: impact on quality of life (parental repots)	**X**		**X**	**X**	**X**			**X**
**Close-out**								
Feedback to each participant				**X**				**X**

Participants in both groups will be advised to book appointments according to treatment needs post each intervention using “Mawid”

### Randomization

A stratified random sampling technique will be performed where schools are the unit of randomization, and stratum will be based on the area of residence to affluent and less-affluent areas. Random sampling will be used to allocate schools to either an intervention or a control group within each stratum. An external researcher will perform the allocation sequence using a computer-generated random number using python program application and then inform the principal investigator (HA), who subsequently will assign the intervention team (performing virtual supervised tooth brushing) to the children in the intervention group.

### Blinding

Children participating in this study cannot be blinded given the nature of the intervention. Both children and their parents will be provided an information sheet that explains the intervention thus will know whether they will be in either intervention or control group. Examiners will be blinded as children in both groups will be dealt with identically in the examination appointment. All outcome assessment data will be delivered to the principal investigators, and thus, each child will be identified to the allocated group according to the child’s code and school code. It is worth mentioning that we chose the caries experience to be the primary outcome as clinical outcomes are considered an objective finding and thus more reliable and less prone to measurement bias associated with subjective self-reported measures.

### Data collection

The study will acquire approval from the Ministry of Health in Saudi Arabia to ensure no school-based dental program is implemented in the selected schools and to utilize the ministries staff and information technology facilities (Mawid, Sehhaty, and ANAT applications). Also, approval from the Ministry of Education will be obtained to both access schools and to obtain a list of schools in Riyadh city.

Following obtaining the approvals, the principal investigators will send an invitation to the selected schools. Schools that refuse to participate or do not reply will be replaced with randomly chosen new school from the same list obtained. For schools that agree to participate, the principal investigator will arrange a date and time for data collection by meeting with the head of the schools.

All children eligible to participate in the schools that agree to participate will receive a booklet that includes an information sheet a written and consent form to both children and their parents. A text message from the school administration will be sent on the same day informing the parents about the booklet and encouraging them to participate. After 1 week, a reminder text message will be sent to parents who did not send the forms back and an acknowledgment text will be sent to those agreeing to participate.

After obtaining signed consent from both parents and their children, the team will book a clinical examination appointment to the children through (Mawid). Parents will receive an SMS message confirming the appointment.

On the day of the booked clinical examination, the parents will be asked to fill structured questionnaire while their children are being examined. The parental questionnaire has been modified from the WHO questionnaire for assessment of oral health status to collect data on sociodemographic characteristics (child’s age and gender, school, household size, parental education and family income), data on dental visit oral hygiene perception and behavior, oral health impact on quality of life, and diet. The questionnaire will be deiminated with every follow-up clinical examination (+ 3 months, + 6 months, + 12 months + 24 months, + 36 months from baseline).

A total number of 40 dental hygienists will be included in this trial under the supervision of the principal investigator. Twenty dental hygienists performing the clinical examination in the Ministry of Health Primary Health Care Centres will be trained and calibrated before the beginning of the trial. The other 20 dental hygienists will be responsible for virtual supervised tooth brushing where two dental hygienists be assigned to each school (2:1 ratio).

Clinical examination for caries experience will be performed according to the WHO criteria that define dental caries at caries into dentine threshold [29]. No radiographs will be taken. Examination will be conducted similarly at baseline and all following follow-ups (+ 3 months, + 6 months, + 12 months + 24 months, + 36 months from baseline). Examination will be performed in a dental clinic on dental chair using an examination kit that includes a plane mouth mirror and a periodontal probe. Teeth will not be brushed or cleaned prior to the examination as plaque score is an outcome of interest. During the examination, air will be applied to better assess the plaque; this will be followed by removal of debris using gauze and cotton roll to facilitate visual inspection and assess gingival score and dental caries. Standard infection control will be in place.

After each clinical examination phase (baseline, (+ 3 months, + 6 months, + 12 months, + 24 months, + 36 months), 10% of children will be re-examined to assess inter-examiner reliability. Children with identified positive caries will be informed, and parents will be instructed to book an online appointment through Mawid. Given the training in oral health condition assessment, the probability of false positive caries cases is minimal. Yet, upon the booked appointment by the parents, a standard dental check-up is always conducted as part of the treatment plan where any false-positive cases will be identified. If that is the case, the dentist will inform the parents that no further treatment is required.

### Ensuring participants’ retention

Given that this is a 36-month trial, with different points of data collection (baseline, + 3 months, + 6 months, + 12 months + 24 months, + 36 months), and the two final follow-ups will be collected over two separate academic years. This implies that there might be some losses in follow-ups as children may change their schools/residences. We have increased the trial size to compensate for 20% attrition rate (i.e., derived from the proportion of children continuing in the same school the following academic year). That aside, we expect a high completion rate as parental consent will be sought for all waves of data collection simultaneously.

### Data storages and management

The principal investigator will be responsible for all data entry and management. Personal identifiable information will be collected to regain contact with the child for the follow-up assessments and link different points of data collected for the same child. This will be handled by a pseudo-anonymization where the principal investigator will keep a separate file that includes all personal identification information for all children participating in the trial. An artificial identifier (code) will be assigned to each child as they enter the study. Thus, all involved in the trial will be working with coded data when collecting or analyzing information.

Only the codes will be used in data entry without any identifiable personal information. A protected device (university desktop) assigned to the principal investigator will be used to enter the coded data as long as the data collection progresses. It is worth noting that consents, assents, and questionnaires will be collected electronically, whereas oral assessment forms will be filled manually through investigators. All electronic forms and questionnaires will be collected through Microsoft Forms empowered by KSU online drive and will be stored in the same drive. Upon completion of the data collection, all electronic files (consents, Assents, and questionnaires) will be transferred to a password-protected desktop computer, and all printed forms (oral assessment forms) will be transferred to a locked cabinet and both located at the College of Applied Medical Sciences at KSU. Only the authorized team members will grant access to the study data.

### Data analysis justification of sample size

Sample size was calculated based on the prevalence of untreated dental caries (84%) reported in a previous study by Adam and his colleagues (2022) among Riyadh children [31]. Considering a 95% confidence interval and 80% power, 690 children (354 individuals per arm) were sufficient to detect a clinically significant difference of 10% with design effect of two arms over a period of 36 months. The design effect was calculated as 1.44, based on an intra-class correlation coefficient of 0.02 derived from a previous study [32]. After accounting for clustering, the required number of clusters would be 20 schools and an average cluster size of 50 participants. Allowing for up to 20% dropout rate upon follow-up time, 1192 participants are required (596 per arm) [33].

### Between groups comparisons

Stata version 17 will be employed for data analysis. In a comparison of demographic (sex, gender), socioeconomic (family income, parental education), behavioral characteristics (tooth brushing frequency, usage of fluoride toothpaste, dental attendance pattern, impact of oral health on daily living, sugar intake), and caries, plaque and gingival level in both primary and permanent teeth, the difference between both intervention groups will be carried out using chi-square test for categorical variables and a *t*-test for continuous variables.

We will be reporting absolute and relative measure effect, that is, absolute and relative difference in mean for continuous outcomes (changes in the number of untreated caries) and risk difference and relative risk for categorical outcomes (changes in brushing frequency, changes in plaque and gingival conditions, changes in impact of oral health on daily living). For the primary outcome (changes in the number of untreated dental caries), negative binomial regression will be used to estimate relative difference (incidence rate ratio) and predictive margin to estimate absolute difference between intervention groups. The applied analytical technique will allow accounting for stratification of the sample and clustering of children within schools and any baseline socioeconomic difference between intervention groups. For the secondary outcome, multinomial regression will be used to estimate relative risk for the changes in frequency of daily tooth brushing and changes in the perceived oral health on child’s daily life. Predictive margins will also be used to estimate absolute difference in each secondary outcome. Intention-to-treat analysis will be used to handle missing values in all outcomes.

## Discussion

Despite the availability of free dental services in Saudi Arabia, dental caries is still a common disease among children. Many studies have focused on the implementation of school-based programs to promote and tackle the burden of untreated dental caries [34]. This study will take a step further and explore the effectiveness of virtual supervised tooth brushing on untreated dental caries especially since Saudi Arabia has proven to have an effective IT infrastructure for virtual schooling.

Three different outcome measures are used to evaluate the effectiveness of virtual supervised tooth brushing on caries experience. The study’s primary outcome is the change in caries experience—the number of decay (untreated dental caries) missing and filled teeth—in both primary and permanent teeth over 36 months (baseline, + 3 months,+ 6 months + 12 months,+ 24 months, + 36 months from baseline) using the WHO criteria [29]. The secondary outcome will be obtained through both clinical outcomes (plaque and gingival scores) and the self-reported questionnaire to assess the change in the frequency of brushing per day and the impact of oral health on the child’s daily life (QoL) [29]. The selected study design will collect data on participants’ socioeconomic and behavioral factors which will help accounting for baseline differences between the two trial groups.

The findings of this study will provide important information on the impact of virtual supervision on dental caries. A positive impact will be an opportunity for targeting a large portion of the population with a high level of disease as a quarter of the Saudi population is younger than 15 years [1]. A positive finding will also support the continuation of virtual supervised tooth brushing in many schools in other parts of the country. Finally, future research should also investigate the factors that could affect the effectiveness of programs. A national study could provide more details about the effectiveness of the program in many different parts of the country.
